# Reinforcement-Learning-Based Robust Resource Management for Multi-Radio Systems

**DOI:** 10.3390/s23104821

**Published:** 2023-05-17

**Authors:** James Delaney, Steve Dowey, Chi-Tsun Cheng

**Affiliations:** Manufacturing, Materials and Mechatronics, School of Engineering, STEM College, RMIT University, 124 La Trobe St., Melbourne, VIC 3000, Australia

**Keywords:** resource management, reinforcement learning, adaptive systems, multi-radio

## Abstract

The advent of the Internet of Things (IoT) has triggered an increased demand for sensing devices with multiple integrated wireless transceivers. These platforms often support the advantageous use of multiple radio technologies to exploit their differing characteristics. Intelligent radio selection techniques allow these systems to become highly adaptive, ensuring more robust and reliable communications under dynamic channel conditions. In this paper, we focus on the wireless links between devices equipped by deployed operating personnel and intermediary access-point infrastructure. We use multi-radio platforms and wireless devices with multiple and diverse transceiver technologies to produce robust and reliable links through the adaptive control of available transceivers. In this work, the term ‘robust’ refers to communications that can be maintained despite changes in the environmental and radio conditions, i.e., during periods of interference caused by non-cooperative actors or multi-path or fading conditions in the physical environment. In this paper, a multi-objective reinforcement learning (MORL) framework is applied to address a multi-radio selection and power control problem. We propose independent reward functions to manage the trade-off between the conflicting objectives of minimised power consumption and maximised bit rate. We also adopt an adaptive exploration strategy for learning a robust behaviour policy and compare its online performance to conventional methods. An extension to the multi-objective state–action–reward–state–action (SARSA) algorithm is proposed to implement this adaptive exploration strategy. When applying adaptive exploration to the extended multi-objective SARSA algorithm, we achieve a 20% increase in the F1 score in comparison to one with decayed exploration policies.

## 1. Introduction

Multi-radio or multi-RAT (multi-radio access technology) systems allow exploitation of the diverse characteristics present in different wireless radio standards, which provides multiple advantages in power consumption and robust link maintenance [1,2,3]. Applications of the multi-radio paradigm are prevalent in two distinct categories: wireless sensor network (WSN)-focused scenarios and heterogeneous access for mobile networks [4,5].

In this work, we consider multi-radio mobile nodes (MNs) deployed in an unknown environment and attached to, or carried by, field operatives that must transmit sensory data to remote command and control centres. As the operating environments are likely to have limited usable communications infrastructure, nodes deployed as local base stations, referred to as stationary nodes (SNs), provide a high-powered back-haul or trunk communications link to a wider area network and subsequently to a command and control centre.

We primarily consider the application of this work in an emergency relief and recovery scenario. Field operations or rescue personnel would be deployed in a remote location (e.g., firefighting teams) to combat imminent fire threats and perform rescue and recovery operations in remote residential areas. A centralised command and control centre manages the coordinated efforts of the operation, monitoring situational awareness information from deployed teams and units, receiving health and equipment monitoring, location tracking, and potentially visual sensory data. With SNs deployed as multi-radio-capable base stations attached to deployment vehicles, the field operatives can move through their local area carrying a multi-radio-enabled personal monitoring and communication device (i.e., an MN that transmits sensory information to an SN). The MN device may be handheld, similar in size to a mobile phone, and have visual, audio, location tracking, or personal health monitoring sensors.

Robust communications [6] and optimised power consumption [7] are critical objectives considered for multi-radio implementations. As such, these themes form the basis of the approach presented in this work. Moreover, we emphasise reinforcement-learning (RL) techniques for intelligent radio selection and transmit power control. Related works show that intelligent techniques are essential for managing the impacts of dynamic channel conditions, which may not always be directly measurable with transceiver hardware.

Q-learning is used in [8] to introduce range adaptability to WSN devices with dual long- and short-range radios. The authors investigated the adaptation to channel conditions for maintaining communication links. They applied a fixed rate of random exploration to discover available lower-power states that achieved a higher immediate reward.

Chincoli and Liotta [7] also employed Q-learning but for transmission power control of a single radio. The reward function proposed was a combination of discrete power levels and a linearly quantised packet reception rate. Similarly to [8], the exploration rate is decayed over time to a minimum value and then fixed for all further iterations. Fixed parameters for exploration rates are also used by Yan et al. [6] and Wang et al. [9]. These works focus primarily on the convergence to optimal solutions rather than online learning performance. However, limited attention is paid to the rate at which learning occurs and whether such approaches are suitable for use in mobile-node-centric radio selection. Although the previous works employed RL methods for learning multi-radio selection or transmit power control, each uses fixed or schedule-based algorithms for managing the exploration and exploitation trade-off [10]. As such, further investigation is required to understand whether the online performance of RL-based methods can be improved through alternative exploration techniques. The online learning performance is tightly bound to the agent’s ability to maximise the reward under environmental dynamics, and in doing so learn optimal policies that satisfy the conflicting objectives of the target application.

In this paper, the effects of exploration on policy behaviour for multi-objective multiradio communications in a dynamic environment are examined. An adaptive strategy is adopted to improve the online performance of multi-radio, multi-objective agents. This strategy is implemented by extending the linear scalarised SARSA algorithm [10], accounting for multi-objective preferences in the adaptive exploration rates. We then apply the multi-objective reinforcement learning (MORL) framework to the multi-radio problem and design reward functions to manage the trade-off between the conflicting requirements of minimising the power consumption and maximising the bit rate. The remainder of this paper is structured as follows: In Section 2, we introduce the simulated environmental model and the MORL framework. In Section 3, we outline the design of the MORL agent and the proposed extended multi-objective SARSA algorithm. Section 4 studies the proposed exploration method’s performance against conventional methods, followed by concluding remarks in Section 6.

## 2. Methodology

The proposed system consists of two logical entities. The first is the wireless communication environment, and the second is the multi-objective reinforcement learning (MORL) agent operating in that environment. In this section, we briefly cover the necessary system model and implementation details, followed by the simulation environment and analytical framework.

### 2.1. Multi-Radio Environment Model

The multi-RAT wireless nodes of focus are equipped with both 2.4 GHz IEEE802.11b (WiFi) 11 Mbps and 915 MHz IEEE802.15.4 250 Kbps transceivers. A combined link layer manages the communication over each interface. The environment consists of both SNs and MNs. It is assumed that the SNs are base stations deployed in a localised area serving multiple MNs. All nodes support two-way traffic and the SNs are considered gateways to back-haul traffic to and from remote control centres. While an MN must select transmission on only one of its available radios, an SN can support concurrent connections to MNs across different RATs.

**Figure 1:**
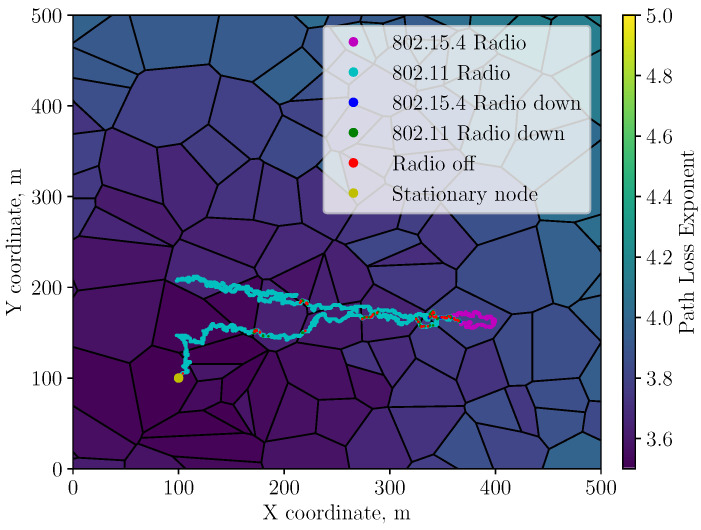
Generated environment and radio selection along the ’linear return’ trajectory.

The physical environment is modelled as a 500 m × 500 m space divided into cells of $1\;m^{2}$. Any node within the space may only occupy a single cell at any time, and without loss of generality, it is assumed that no two nodes may concurrently occupy the same cell. Channel conditions are modelled using the log-distance propagation model, expressed as

(1) $$P_{r,\mathrm{SN}}=P_{t,\mathrm{MN}}-PL\left(d_{0}\right)-10\gamma \log_{10}\left(d/d_{0}\right)+X,$$

where $P_{r,\mathrm{SN}}$ is the received power at the SN in dBm and $P_{t,\mathrm{MN}}$ is the transmit power in dBm of the MN. For simplicity, all antennas are assumed to have unity gain ($G_{t}$ and $G_{r}=0\;\mathrm{dB}$). $PL(d_{0})$ is the free space path loss at a reference distance $d_{0}$ of 10 m. Fluctuations in the channel conditions are simulated by artificially dividing the space into zones using a Voronoi process with a random distribution of centroids as shown in Figure 1. The path-loss exponent within a zone is assumed to be uniform and is calculated based on the distance between its centroid and the SN. The path-loss exponent γ and log-normal shadowing process *X* in (1) model the dynamic channel conditions. The trajectory of the MN follows a Gauss–Markov random walk with the MN moving at 3–5 km/h. According to Camp et al. [11], the corresponding speed and direction are updated using

(2) $$s_{t}=\alpha s_{t-1}+\left(1-\alpha \right)\bar{s}+\sqrt{\left(1-\alpha^{2}\right)s_{x_{t-1}}},$$

(3) $$d_{t}=\alpha d_{t-1}+\left(1-\alpha \right)\bar{d}+\sqrt{\left(1-\alpha^{2}\right)d_{x_{t-1}}},$$

where $s_{t-1}$ and $d_{t-1}$ are the speed and direction at the previous time step and α is a tuning parameter. Here, $\bar{s}$ and $\bar{d}$ respectively represent the mean speed and direction of the MN, and $s_{x_{t-1}}$ and $d_{x_{t-1}}$ are Gaussian random variables. The system simulation, environment, and RL algorithm are all updated at intervals of 200 ms (T=0.2s).

### 2.2. Multi-Objective Reinforcement Learning

The multi-objective RL framework differs from its single-objective counterpart in the structure of the reward that an agent receives. In a problem with *N* objectives, the reward function $R\in R^{n}$ returns a vector of *N* rewards with each element corresponding to one objective.

Using the framework in [10], the agent is presented with a representation of the environment’s state at each time step $S_{t}\in \left(S\right)$ and selects a discrete action $A_{t}\in \left(A\right)$ based on the observations and corresponding state representation available. In the following time step, the agent receives a reward vector $R_{t+1}\in \left(R\right)\in R$ from the environment. The rewards are functions of the actions taken and the resulting next-state observations, $S_{t+1}$.

### 2.3. Simulation Environment and Analytical Framework

Using the aforementioned multi-radio environment model and the MORL framework, we have developed a simulation platform to conduct experiments that utilise these theoretical models. The simulation platform has been developed as a collection of Python scripts and makes use of standard numerical and scientific computation and visualisation libraries (namely Numpy [12], Matplotlib [13], and Pandas [14]). We also use the OpenAI gym package [15] and have developed a reinforcement learning environment to model multi-radio wireless devices that utilise simulated mobility in a pre-generated emulation of a physical environment. The gym package also provides an interface for developing RL-based agents to interact with these environments.

To begin each experiment, a certain agent and multi-radio simulation environment configuration are specified. For the agent, this will be the control algorithm that performs selection and power control over an MN operating in a multi-radio scenario. The transceiver parameters used in the simulation are shown in Table 1, while for the environment, this will be the dynamic environment model from Section 2.1, the SN location fixed at a randomised location for a single environment, the pre-generated MN trajectory governed by (2) and (3), and any agent-specific configurations that the environment must manage. These configurations are the MN data application configuration and reward functions for RL-based agents or action space configuration in terms of how the transceiver selection is managed. Once initialised, the control-agent algorithm is started and fed the initial environmental state. The simulation continues in a turn-based manner where the agent takes an action and returns a state observation from the environment and a reward signal before taking another action. This cycle continues for a fixed number of steps resulting from the generated Gauss–Markov trajectory.

The simulation scripts can be run on a desktop computer and require only an installation of Python 3 and the aforementioned libraries. They are computationally inexpensive, which reflects the applicability of the target implementation platforms for the algorithms where they are likely to be applied to low-powered embedded communication platforms.

## 3. The Proposed Solution

### 3.1. State and Action Spaces

The multi-radio RL agent’s state and action spaces take into account the available radios and transmit power levels of the system, reflecting a similar design to that suggested in [8]. The agent’s action space is defined as a set of actions $A=\{a_{i}|i\in I\}$ where I={0,1,⋯,10}. Actions ${\left\{a_{i}\right\}}_{0\leq i\leq 4}$ select one of the 5 discrete power levels in the 2.4 GHz IEEE802.11b radio, where $a_{0}$ is the lowest and $a_{4}$ the highest transmit power. The same holds for the 915 MHz IEEE802.15.4 radio in ${\left\{a_{i}\right\}}_{5\leq i\leq 9}$. The final action, $a_{10}$, accounts for instructing the agent to stop transmitting. The set of states S is aligned with the action space; hence, it is similarly defined as $S=\{s_{i}|i\in I\}$ where I={0,1,⋯,10}. Thus, in terms of state transitions, the action at time *t* will move the agent to the corresponding state at time t+1.

### 3.2. Reward-Function Design

Two reward functions are proposed to obtain a desirable energy and goodput trade-off. Each reward function is designed such that the agent is rewarded for maintaining link-level connectivity or ceasing to transmit where no link is available.

The rewards for each objective are defined as conditional functions. This provides the agent with the ability to learn generalised policy behaviour without being directly instructed on how to behave under certain conditions. Generality in the policies is achieved by imparting domain knowledge and designing the reward functions such that they are dependent on the environmental conditions (i.e., signal strength and link status). The advantage is that the agent will learn a policy that is dependent on the dynamics of the environment rather than a fixed set of behaviours, thus allowing the agent to adapt to new conditions within an environment or even new environments. We also use shaping techniques that are shown to accelerate learning without impacting the quality of policy that is learned [18].

As is defined by the MORL framework, the agent receives a vector of rewards at each step, where each element corresponds to the reward returned by the functions defined for each objective. Consequently, the agent now has the capability to learn from multiple sources of reward. As such, the agents receive higher, more positive rewards for selecting actions that better address the requirements of that objective. However, they are penalised by negative rewards when their actions deviate from desirable behaviours. The values for each reward function are always scalar and fall within the range [−1,1]. There are two caveats to this specification:

1. Positive rewards are only given for actions that result in a successful link between MN and SN.
2. In the event that no link can be established on any transceiver, the agent should choose not to transmit.

In the case that neither of these conditions is met, the agent is penalised with a negative reward value.

#### 3.2.1. Objective 1. Maximised Bit Rate

Under this objective, a higher reward is gained for transmitting on a radio with a higher bit rate. The reward function is defined as follows

(4) $$r_{b}\left(B_{t},B\right)=\left\{\begin{array}{cc} 1, & \mathrm{case}\;1\\ -1, & \mathrm{case}\;2,\;3\\ \frac{B_{t}-min\left(B\right)}{max(B)-min(B)}, & \mathrm{otherwise} \end{array}\right.,$$

**Case 1:** if no link is available on any radio and $a_{t}=a_{10}$.

**Case 2:** if a higher-bit-rate radio is available.

**Case 3:** if the link is down on the selected radio.

The rewards for all cases other than those described are assigned based on the unit normalised bit, rate where $B_{t}$ is the bit rate of the current radio and B is the set of available radios’ bit rates. This structure encourages the agent to choose a higher-bit-rate radio when possible. It also ensures that a positive but attenuated reward is given to the agent when the selected radio does not have the highest bit rate.

#### 3.2.2. Objective 2. Minimised Power Consumption

This objective follows a pattern where the reward is higher for utilising lower power states of the selected radio. The reward function for minimising power consumption is structured such that it will drive the agent to select the lowest available power level while maintaining a link. This is achieved through a similar shaping approach as in Section 3.2.1 and $r_{p}$ in (5) is subject to a number of cases based on the state of all radios and the selected power level. Here, $P_{tx}$ is the current power level and $P_{\mathrm{tx}}$ is the set of available transmission power levels.

(5) $$r_{p}\left(P_{tx},P_{\mathrm{tx}}\right)=\left\{\begin{array}{cc} 1, & \mathrm{case}\;4\\ -1, & \mathrm{case}\;5\\ -\frac{P_{tx}-min\left(P_{\mathrm{tx}}\right)}{max\left(P_{\mathrm{tx}}\right)-min\left(P_{\mathrm{tx}}\right)}, & \mathrm{case}\;6\\ 1-\frac{P_{tx}-min\left(P_{\mathrm{tx}}\right)}{max\left(P_{\mathrm{tx}}\right)-min\left(P_{\mathrm{tx}}\right)}, & \mathrm{otherwise} \end{array}\right.$$

**Case 4:** if no link is available and the agent selected *not* to transmit.

**Case 5:** if no link is available at the selected power level.

**Case 6:** if a lower power level is available.

### 3.3. WAMO-SARSA Algorithm with Adaptive Exploration

In this section, we present our proposed multi-objective SARSA algorithm with an integrated adaptive exploration strategy (i.e., adaptive VDBE [19]). We extend the multi-objective on-policy SARSA algorithm, GM-SARSA, proposed by Sprague and Ballard in [20], to implement the adaptive VDBE algorithm. The extensions incorporate the current objective preferences into the original algorithm presented by Tokic in [19] for single-objective RL algorithms. This approach ensures that the objective preferences contribute accordingly to adaptive exploration and subsequent action selection. We have named this implementation of the algorithm weighted adaptive multi-objective SARSA (WAMO-SARSA), where the SARSA algorithm is named such that its update rule uses the tuple $\left(s_{t},a_{t},r_{t},s_{t+1},a_{t+1}\right)$. The WAMO-SARSA algorithm is shown in Algorithm 1 for the general case of *N* objectives.**Algorithm 1:** Weighted Adaptive Multi-Objective SARSA (WAMO-SARSA).
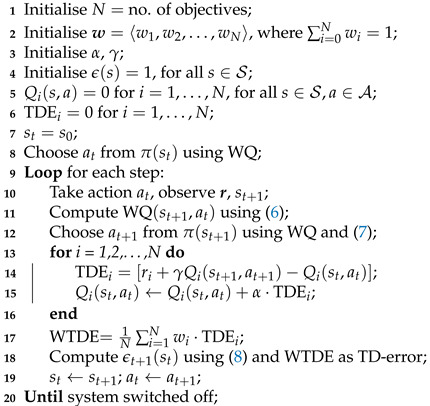


First, the input parameters α and γ are the learning rate and discount factor, respectively. The learning rate controls how quickly the value of an action can increase/decrease with rewards received at each iteration of the algorithm, while the discount factor governs the influence of these immediate rewards at each step. That is to say, a lower discount factor results in more adaptive behaviour because an agent will tend to learn higher action values for actions that produce an immediate reward rather than for those that result in longer-term gains. The other input parameters, *N* and ω, are directly, related as *N* determines the size of ω. It should be stated that ω are the weights of each objective, where each reward function is designed to address a different objective.

We will describe the proposed extensions in terms of differences to GM-SARSA in [20], outlining the contributions made to incorporate the adaptive VDBE method. Compared with GM-SARSA, it can be seen in Algorithm 1 that there are changes and additions at lines 4, 6, 12, 14, 15, 17, and 18. First, at line 4, we initialise the VDBE algorithm and define ϵ(s)=1 for all states in the state space according to [19]. Following this, at line 6, we also define ${\mathrm{TDE}}_{i}$ to hold the TD-error for each of the *N* objectives.

(6) $$\mathrm{WQ}\left(s,a\right)=\sum_{i=0}^{N}w_{i}Q_{i}\left(s,a\right),$$

At line 12, we specify ϵ-greedy exploration where state-dependent probabilities are used to select actions. This modified ϵ-greedy algorithm is shown in (7), where ϵ(s) replaces the single value for ϵ with a state-dependent value updated according to (8).

(7) $$\pi \left(s\right)=\left\{\begin{array}{cc} \mathrm{random}\;\mathrm{action}\;\mathrm{from}\;A(s) & \mathrm{if}\;\zeta <\epsilon (s)\\ {\mathrm{argmax}}_{a\in A(s)}\mathrm{WQ}\left(s,a\right) & \mathrm{otherwise} \end{array}\right.,$$

Lines 14 and 15 of Algorithm 1 divide the standard update rule for multi-objective SARSA into two components. For each of the *N* objectives, we calculate the TD-error, store it in ${\mathrm{TDE}}_{i}$, and perform an update on the respective action-value function for the ith objective (i.e., $Q_{i}\left(s_{t},a_{t}\right)$). Then, using the stored TD-errors across all objectives, we define the weighted TD-error (WTDE) at line 17. This WTDE quantity is the sum of TD-errors at the current step weighted by each of the corresponding objective weights in ω. Lastly, we compute the next value for $\epsilon_{t+1}\left(s_{t}\right)$ in the current state $s_{t}$, substituting WTDE for the TD-error in (8). Recall that the original adaptive VDBE algorithm for computing $\epsilon_{t+1}\left(s_{t}\right)$ is

(8) $$\epsilon_{t+1}\left(s\right)=\delta \cdot \frac{1-e^{\frac{-|\mathrm{TD}-\mathrm{error}|}{\sigma_{e}}}}{1+e^{\frac{-|\mathrm{TD}-\mathrm{error}|}{\sigma_{e}}}}+\left(1-\delta \right)\cdot \epsilon_{t}\left(s\right),\delta =\frac{1}{\left|A(S)\right|},$$

where $\sigma_{e}$ controls how much ϵ changes based on the TD-error at each time step and δ is always set to the inverse of the number of actions in the action space [21]. Here, the parameter $\sigma_{e}$ is referred to as the inverse sensitivity, lower values of which cause high rates of exploration (i.e., ϵ(s) closer to 1) when the magnitude of the TD-error is high, noting that ϵ(s) is initially set to 1 for all states (total exploration). Then, as the agent interacts with the environment, the relation in (8) causes ϵ(s) to reduce as the TD-error moves closer to zero for each state *s* (as the agent learns to act optimally). When environmental dynamics occur and the TD-error increases as a result of the reward functions following these dynamics, ϵ(s) will increase and encourage the agent to begin exploring for more optimal actions.

In summary, our proposed method replaces the TD−error in (8) with WTDE, a weighted summation of the TD-errors for the action-value function corresponding to each objective at the current step, thus making the adaptive VDBE strategy now dependent on objective preferences and ensuring that action selection is also equally dependent on these same preferences.

Using these extensions, we are now able to effectively implement an adaptive exploration strategy for a multi-objective RL agent.

## 4. Results

In this section, we study the behaviour of the RL agent with respect to the objectives previously defined (N=2) and two exploration strategies. The weights of the reward functions $r_{b}$ and $r_{p}$ are $w_{1}$ and $w_{2}$, respectively, with both set to 0.50 for a balanced trade-off between objectives. This weighting drives the agent to choose a power level as low as possible while using the highest-bit-rate radio possible. Learning algorithm parameters are kept constant for all experiments where α=0.7 and γ=0.1. The inverse-sensitivity parameter σ for VDBE is set to 0.08. The path-loss exponent in the Voronoi regions in Figure 1 is varied between 3.5 and 5.0, where each trajectory will encounter different dynamics within the same environment.

The values for α and γ were selected as they yielded the highest F1-score across a range of objective weights during testing. As we do not consider the direct effects of modifying these parameters in this paper, we have also selected these values to suit the application scenario for adaptive MR communications. In simple terms and with reference to the discussion of the impact of these parameters in Section 3.3, we are interested in an agent that favours immediate rewards highly and also one that is able to quickly converge to stable action values.

### 4.1. Adaptive Exploration Policy Evaluation

The online learning performance of the WAMO-SARSA agent was investigated with two exploration strategies. The first strategy reduced the exploration parameter over time to a minimum value as used in [7,8] and is referred to as the decayed exploration rate. The second was the multi-objective VDBE proposed in Section 3.3, which is referred to as the adaptive exploration rate.

**Figure 2:**
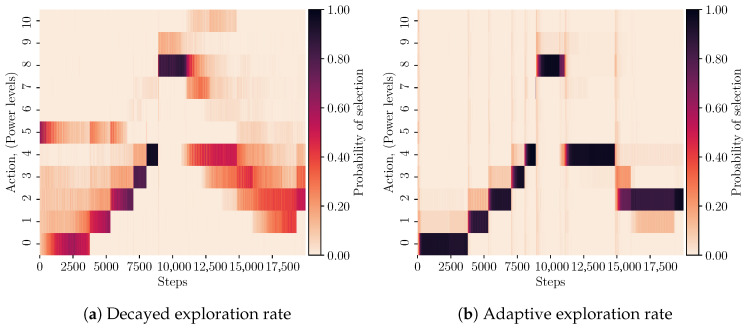

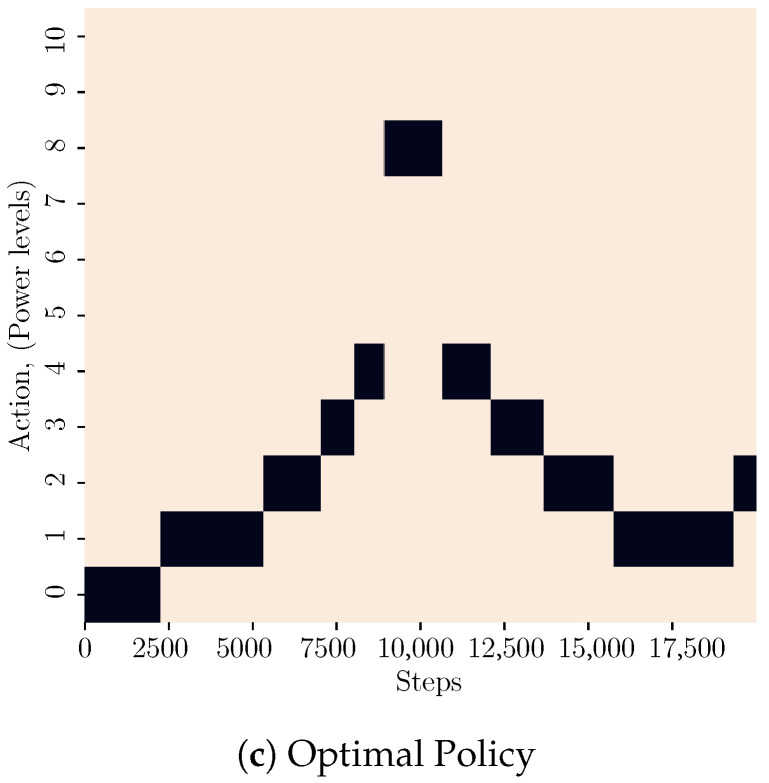
WAMO-SARSA policy behaviour for the ‘linear return’ trajectory. In (**b**) the agent learns a higher-consistency policy than in (**a**). This is reflected by the precision measure in Table 2, where the precision for adaptive and decayed exploration is 0.72 and 0.62, respectively. The optimal policy is shown in (**c**).

A sample learned policy of the WAMO-SARSA agent for both exploration strategies is shown in Figure 2a,b. The figures plot the average performance over 100 runs of radio and power-level selection against steps along the trajectory. The average performance is indicated via heat maps, where the intensity indicates the relative likelihood of selecting a particular action at each step along the trajectory. Figure 2c shows the optimal policy that achieves the maximum reward at every step. Recalling from Section 3.1, actions ${\left\{a_{i}\right\}}_{0\leq i\leq 4}$ select the IEEE802.11b radio and associated power levels while actions ${\left\{a_{i}\right\}}_{5\leq i\leq 9}$ select the IEEE802.15.4 radio.

An important difference to note between the performance of each exploration strategy is in the decisiveness and consistency of the learned policies. This is shown by the darker and more defined shapes in Figure 2b that contrast the lighter gradients in Figure 2a. The agent using adaptive exploration rates is less likely to deviate from a single policy when the same experiment is repeated a number of times.

### 4.2. Adaptive Exploration Performance Evaluation

**Figure 3:**
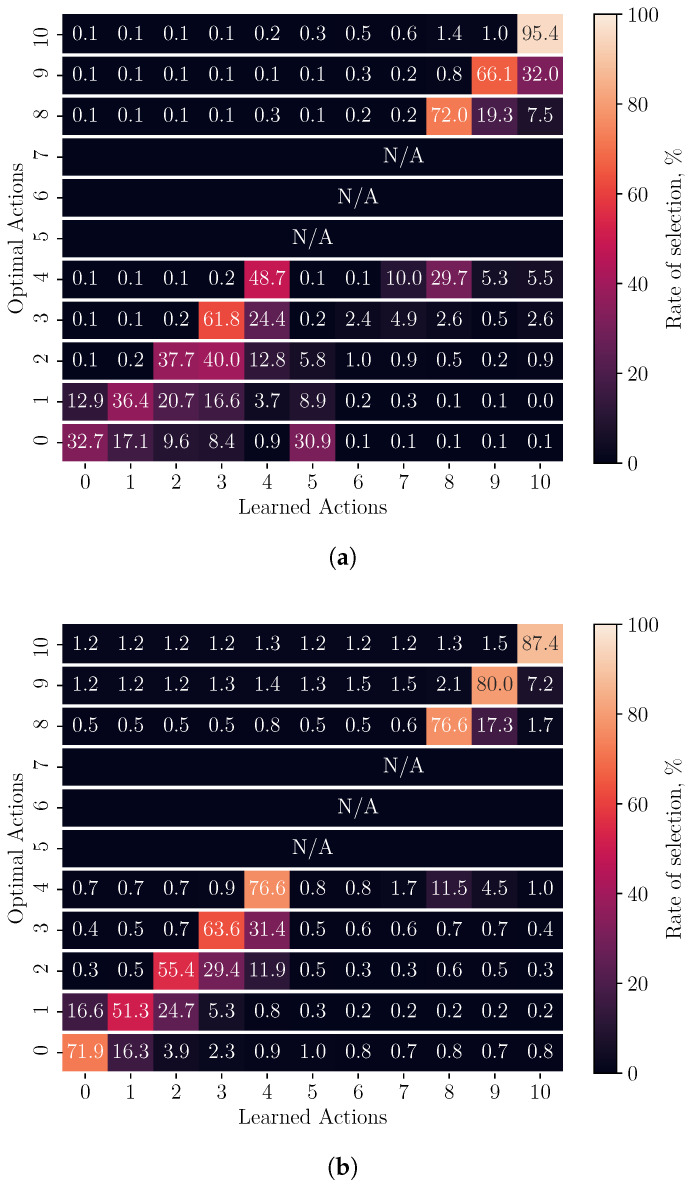
Confusion-matrix representation of policy behaviour for decayed and adaptive exploration techniques in all environments. Diagonal boxes show the rate that a particular action is selected when optimal. Percentages are calculated row-wise and are used to derive the recall rate for each exploration technique. Actions 5, 6, and 7 are not used by the optimal policy in any of the three environments and are shown as ‘N/A’. (**a**) Decayed exploration rate. (**b**) Adaptive exploration rate.

To quantify the policy performance with respect to the optimal policy, we use a confusion matrix to visualise the accuracy and consistency of each exploration method in Figure 3a,b. The learned policy is compared to the *optimal* and each row of the matrix represents the performance with respect to steps where a particular action is optimal. The diagonal regions show the rate at which each action is optimally selected by the agent. The regions on either side of the diagonal demonstrate the likelihood that a non-optimal action is chosen in place of the optimal.

The matrix is constructed by iterating over the optimal policy and recording the learned action in the row corresponding to the optimal action at that step. For example, at a step in which the agent selected $a_{8}$ and the optimal action is $a_{4}$, the count at row 4 column 8 is incremented. This procedure is repeated for all simulation runs across the three environments to derive the rate of selection for each learned action (column) per optimal action (row).

Table 2 shows two measures commonly adopted in the classification literature for each exploration method, namely, precision and recall. A third measure, the F1-score, is used to measure the harmonic mean of precision and recall [22]. Precision indicates the ratio of correctly selected optimal actions in each class of learned action, while recall is the rate that each action is selected optimally and represents the row-wise spread around the diagonal in confusion matrices. We perform macro-averaging to gain a statistic for the overall precision, recall, and F1-score for the action space.

## 5. Discussion

It can be seen in Table 2 that there is a minimum 15% increase in the precision, recall, and subsequent F1-score when an adaptive exploration rate is used over a decayed rate. This can be seen in Figure 3b by the increased selection rates at the diagonal regions and less horizontal distribution on either side of the optimal actions when compared to Figure 3a. This increase is also visible in the differences between policy heat maps for the ’linear return’ environment in Figure 2a,b when compared to the optimal in Figure 2c. The difference in these figures, shown by darker-coloured regions, also demonstrates that the adaptive agent learns a more consistent policy along its trajectory when the experiment is repeated many times. From the communication performance figures in Table 3, the total amount of data that were successfully transferred from MN to SN increased while the packet loss rate decreased when adaptive exploration rates were used over decayed in all environments. Both are indicators of a more stable radio and power-level selection policy.

However, the power consumption under adaptive exploration tends to be higher than that of the decayed exploration agent but closer to the optimum policy when the heat maps in Figure 2a–c are compared. This can be attributed to the nature of the adaptive exploration algorithm where high rates of exploration occur during periods of uncertainty and environmental changes. These are shown by the apparent vertical bars in Figure 2b at the boundary between notable changes in action selection along the trajectory.

While these periods are short on the scale of the total system operation time, it highlights the importance of balancing exploration and exploitation under dynamic channel conditions to reduce the time spent behaving sub-optimally. It is this adaptive and environment-dependent behaviour of the adaptive exploration method that makes it a more suitable approach for learning in these environments than arbitrary schedule-based or fixed exploration rates. There is also a reduced dependence on offline design and experimentation to select the appropriate learning algorithm parameters.

In future works, we will consider approaches to minimise these periods of exploration and further reduce the dependence on offline parameter optimisation (i.e., optimising the parameter σ for adaptive exploration, α and γ for SARSA in Algorithm 1).

With respect to the operation of the WAMO-SARSA algorithm and how it is intended to be implemented, it is designed to be operated on the MN side of the system and is independent of the base stations or SNs. Thus, it is the MNs that perform radio selection and control independently. Their actions are purely governed by the performance seen by each MN with respect to its corresponding SN in the network. Hence, the algorithm is largely unaffected by a scaled wireless network in terms of nodes. However, the processing power and ability of a base station to communicate with many nodes will be a limiting factor, as with any conventional network scheme. While scaling the number of objectives will increase the granularity with which control over radio selection can be imparted, there is a risk that this will create a watered-down effect of each objective on the system’s behaviour. Although not considered here, in future works we will also investigate how the number of objectives would impact learning abilities.

Lastly, we will briefly examine the algorithm in terms of computational complexity and memory requirements. It is intended that the algorithm proposed in this paper be implemented using low-powered and embedded devices that have integrated wireless communications modules. The WAMO-SARSA algorithm does not involve complex signal processing or time-consuming iterative calculation at each iteration. Rather, each iteration involves a series of linear operations that are scalarised across multiple objectives without the need for computations that directly use matrices or vectors. Further to this, the scalarisation of action values across objectives is only completed for a single action-value pair per iteration, not the entire state and action spaces. Thus, we believe that the algorithm is appropriate for targeting these embedded systems that utilise RISC (Reduced Instruction Set Processors), such as the ARM Cortex-M series of processors. However, it is also important to consider how the algorithm performs with respect to the number of objectives regardless of the relative simplicity of computations required. As a separate table of action values (*Q*) is stored for each objective, intuitively we can see that the memory required would scale linearly with the number of objectives. However, as previously discussed, it may be preferable to keep the number of objectives limited to ensure that the ability of different communication objectives to be successfully achieved is maintained.

## 6. Conclusions

This paper has investigated the use of the MORL framework for adaptive radio selection and power control in multi-radio wireless systems. We have applied adaptive policy exploration methods for multi-objective RL agents to the MR-selection problem, where previous studies [6,8,9] have only examined or applied exploration policies with decaying rates of exploration. To perform this comparison we additionally proposed an extension to the GM-SARSA algorithm [20]. Independent reward functions have been designed to provide an effective trade-off between conflicting objectives, and an extension to multi-objective SARSA was proposed for an adaptive exploration strategy. The results show that the proposed extended algorithm learns a policy that outperforms conventional exploration methods, achieving a 20% increase in the F1-score across three varied environments and mobility traces. These performance gains demonstrate that adaptive exploration is a more suitable candidate for online learning and control in multi-radio, multi-objective wireless communication systems.

## Figures and Tables

**Table 1:** Transceiver parameters used in the simulation.

Parameter	IEEE802.11b [16]	IEEE802.15.4 [17]
Operating voltage (V)	3.3	3.3
Frequency (MHz)	2400	915
Bandwidth (MHz)	20	2
Bitrate (Kbps)	11,000	250
Modulation	BPSK	QPSK
Receiver sensitivity (dBm)	−97	−110
MTU (bytes)	2346	127

**Table 2:** Exploration method precision, recall, and F1-score comparisons evaluated across all three environments.

	Decayed	Adaptive
Precision	0.62	0.72
Recall	0.56	0.70
F1-score	0.59	0.71

**Table 3:** Exploration method communication-performance comparison.

Environment	Data Tx, MB		PLR, %		Power, Wh	
	**Decayed**	**Adaptive**	**Decayed**	**Adaptive**	**Decayed**	**Adaptive**
Linear return (Figure 1)	103.04	110.21	15.77	3.40	0.50	0.58
Far boundary	64.82	75.42	25.31	11.21	0.24	0.32
Near return	112.85	116.42	7.25	1.57	0.58	0.59

## Data Availability

Not applicable.

## Abbreviations

The following abbreviations are used in this manuscript:

BPSK	Binary Phase Shift Keying
GM-SARSA	Greatest-Mass Multiple-Goal Reinforcement Learning with Modular SARSA
IoT	Internet of Things
MN(s)	Mobile Node(s)
MORL	Multi-Objective Reinforcement Learning
Multi-RAT	Multi-Radio Access Technology
QPSK	Quadrature Phase Shift Keying
RL	Reinforcement Learning
SARSA	State–Action–Reward–State–Action
SN(s)	Stationary Node(s)
TD	Temporal Difference
VDBE	Value-Difference-Based ϵ-greedy Exploration
WAMO-SARSA	Weighted Adaptive Multi-Objective SARSA
WSN(s)	Wireless Sensor Network(s)
WTDE	Weighted TD-Error
